# Western diet consumption by host vertebrate promotes altered gene expression on *Aedes aegypti* reducing its lifespan and increasing fertility following blood feeding

**DOI:** 10.1186/s13071-023-06095-3

**Published:** 2024-01-06

**Authors:** Alexandre Menezes, Marilia Peixoto, Melissa Silva, Emylle Costa-Bartuli, Cinara Lima Oliveira, Ana Beatriz Walter-Nuno, Nathan da Cruz Kistenmacker, Jessica Pereira, Isabela Ramos, Gabriela O. Paiva-Silva, Geórgia C. Atella, Patricia Zancan, Mauro Sola-Penna, Fabio M. Gomes

**Affiliations:** 1 Laboratório de Ultraestrutura Celular Hertha Meyer, Universidade Federal do Rio de Janeiro, Rio de Janeiro, Brazil; 2 The Metabolizsm’ Group, Departamento de Biotecnologia Farmacêutica, Faculdade de Farmácia, Universidade Federal do Rio de Janeiro, Rio de Janeiro, Brazil; 3 Laboratório de Bioquímica de Lipídeos e Lipoproteínas, Instituto de Bioquímica Médica Leopoldo De Meis, Universidade Federal do Rio de Janeiro, Rio de Janeiro, Brazil; 4 Laboratório de Bioquímica e Biologia Molecular de Artrópodes Hematófagos, Universidade Federal do Rio de Janeiro, Rio de Janeiro, Brazil; 5 Instituto Nacional de Ciência e Tecnologia em Entomologia Molecular, Rio de Janeiro, Brazil; 6 Laboratorio de Ovogênese Molecular de Insetos Vetores, Universidade Federal do Rio de Janeiro, Rio de Janeiro, Brazil

**Keywords:** Metabolic syndrome, Western diet, Malnutrition, Immunometabolism, *Aedes aegypti*, Vector capacity, Vector competence, Blood feeding

## Abstract

**Background:**

The high prevalence of metabolic syndrome in low- and middle-income countries is linked to an increase in Western diet consumption, characterized by a high intake of processed foods, which impacts the levels of blood sugar and lipids, hormones, and cytokines. Hematophagous insect vectors, such as the yellow fever mosquito *Aedes aegypti*, rely on blood meals for reproduction and development and are therefore exposed to the components of blood plasma. However, the impact of the alteration of blood composition due to malnutrition and metabolic conditions on mosquito biology remains understudied.

**Methods:**

In this study, we investigated the impact of whole-blood alterations resulting from a Western-type diet on the biology of *Ae. aegypti*. We kept C57Bl6/J mice on a high-fat, high-sucrose (HFHS) diet for 20 weeks and followed biological parameters, including plasma insulin and lipid levels, insulin tolerance, and weight gain, to validate the development of metabolic syndrome. We further allowed *Ae. aegypti* mosquitoes to feed on mice and tracked how altered host blood composition modulated parameters of vector capacity.

**Results:**

Our findings identified that HFHS-fed mice resulted in reduced mosquito longevity and increased fecundity upon mosquito feeding, which correlated with alteration in the gene expression profile of nutrient sensing and physiological and metabolic markers as studied up to several days after blood ingestion.

**Conclusions:**

Our study provides new insights into the overall effect of alterations of blood components on mosquito biology and its implications for the transmission of infectious diseases in conditions where the frequency of Western diet-induced metabolic syndromes is becoming more frequent. These findings highlight the importance of addressing metabolic health to further understand the spread of mosquito-borne illnesses in endemic areas.

**Graphical Abstract:**

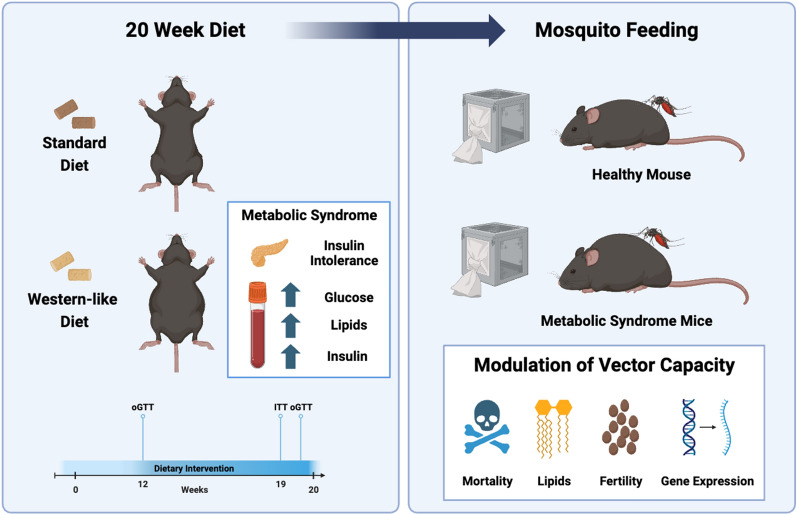

**Supplementary Information:**

The online version contains supplementary material available at 10.1186/s13071-023-06095-3.

## Background

The World Health Organization has identified obesity as one of the most serious public health challenges of the twenty-first century, with > 650 million adults worldwide being classified as obese [1]. This epidemic is largely driven by the global increased consumption of Western diets [2], characterized by a high intake of processed foods, added sugars, unhealthy fats, and a low intake of fruits, vegetables, and whole grains [3]. In low- and middle-income countries (LMIC), Western dietary patterns often correlate with economic transition in high socioeconomic families [4] and the increased affordability of cheap, calorie-dense processed foods compared to fresh, whole foods in the lower socioeconomic strata [5, 6]. These dietary habits result in alterations of blood composition, including levels of inflammatory cytokines, lipids, glucose, and other blood components. As such, Western diets are predisposing factors for developing obesity, type 2 diabetes, and other non-communicable diseases, such as cardiovascular diseases—the leading cause of death globally [7]. The simultaneous occurrence of these conditions is defined as metabolic syndrome [8].

Mosquito-borne illnesses like malaria, transmitted by *Anopheles *sp., or dengue, and Zika, primarily transmitted by *Aedes aegypti*, are major threats in tropical LMIC areas of the world [9]. The transmission of these diseases is strictly linked to the ingestion of blood by mosquitoes. This is a necessary step for pathogen development in the mosquito host and transmission following the pathogen cycle in the mosquito. Blood ingestion and digestion trigger nutrient sensing pathway-dependent physiological response [10–12] that must be tightly coordinated through several organs to coordinate energetic balance and immune status [13]. Mosquito vitellogenesis is also coordinated during this process. Here, a blood meal triggers a 20-hydroxyecdysone (20E) hormonal cascade [14], which drives the expression of the yolk protein precursor gene vitellogenin under the control of target of rapamycin (TOR) [15] and insulin signaling pathways [16]. Concurrently, the lipid transport protein lipophorin [17] mobilizes triglycerides [18] from the fat body toward the ovaries.

Previous studies have highlighted that individual components of the blood plasma can regulate this balance and influence vector biology and immunity. For example, it has been shown that human insulin and IGF1 supplementation during artificial meals can activate AKT and ERK-dependent pathways and reduce mosquito longevity [19] and immunity [20, 21]. A negative impact on *Plasmodium* infection levels has also been observed in *Anopheles* fed with a TGFß-1 supplemented blood meal [22]. In *Aedes*, other blood components, such as glucose, have also been shown to modulate mosquito vector capacity through AKT/TOR [23]. However, artificial single-point alterations of blood composition rarely reflect the blood's physiological modulations where several components are found altered because of metabolic syndromes and altered physiological states [24, 25]. In that sense, the impact of metabolic syndromes and nutritional imbalance, such as those derived from Western-type diets on mosquito biology, remains mostly unknown.

In this study, we investigated how blood alterations derived from a Western diet affect *Aedes aegypti* biology and several components of vector capacity. To do this, we fed mice a high-fat high-sucrose (HFHS) diet for 20 weeks, inducing weight gain, insulin resistance, and other metabolic syndrome-related conditions, and allowed mosquitoes to feed on these animals. Our findings identified an impact on mosquito longevity and fitness because of the ingestion of blood from mice with metabolic syndrome. Albeit a discrete impact on the overall transcriptional profile, we identified markers that were modulated because of the vertebrate host metabolic status. Our study provides new insights into the overall effect of alterations of blood components on mosquito biology and may have implications for the transmission of infectious diseases in conditions where the frequency of Western diet-induced metabolic syndromes is becoming more frequent. These findings highlight the importance of addressing metabolic health to further understand the spread of mosquito-borne illnesses.

## Methods

### *Aedes aegypti* rearing

The *Ae. aegypti* Red Eye strain used in this study has been kept at Universidade Federal do Rio de Janeiro since 2000. For this study, 100–150 larvae were reared in 1L of filtered water at 27 ± 1 °C with a 12 h:12 h light:dark cycle. Larvae were fed 1.5 g of Pedigree dog chow. Pupae were collected and placed in a 3-L plastic cage for adult emergence, maintained at 27 ± 1 °C and 70% relative humidity with a 12 h:12 h light:dark cycle. Adults were fed a 10% sucrose solution and allowed to mate in the cage before being used for experiments.

### Mice diet

Six-week-old C57Bl6/J mice were used and were kept under controlled conditions (24 ± 1 °C; 55 ± 15% humidity; and 12 h/12 h light/dark cycle) during the 20-week protocol. After the acclimation period, animals were randomly divided into two groups of 40 animals each; one group was given a chow AIN93M diet (control group) and the other to a HFHS diet (HFHS group). The composition of CHOW and HFHS diets [26] is presented in Additional file 1: Table S1. Mice were allowed to feed and drink water ad libitum during the entire protocol.

### Weight monitoring, insulin, and glucose tolerance tests

Throughout the 20-week dietary intervention, animals' body weights were measured weekly using a digital balance. In the 12th and 20th week of the protocol, animals underwent an oral glucose tolerance test (oGTT) to assess their responsiveness to the diet. In the 19th week, an insulin tolerance test (ITT) was performed to evaluate insulin resistance. At the end of the 20 weeks of intervention, the animals were killed, and their serum was collected and stored. The oGTT and ITT were performed as previously described [27]. Briefly, animals were fastened for 5 h before performing the tests. For oGTT, a dextrose solution was administered at a dose of 2 g per kilogram of body weight for each mouse. For ITT, 0.5 U of insulin (Humalin R, Eli Lilly, Indianapolis, IN, USA) per kilogram of body weight for each mouse was intraperitoneally injected. Blood samples were collected from the caudal vein immediately just before the start of the procedure and 15, 30, 60, and 120 min after administration of a single dose of dextrose or insulin. Glycemia was evaluated using a FreeStyle Precision Neo glucometer (Abbott Laboratories, Chicago, IL, USA). To measure liver cholesterol and triglycerides, Folch's method was used as described previously [28], and plasma insulin levels were assessed by ELISA using the Insulin Mouse ELISA Kit (ThermoFisher), following manufacturer instructions. Aspartate aminotransferase (AST) and alanine aminotransferase (ALT) were evaluated as a marker of liver damage, as previously described [29].

### Mosquito feeding on mice

To assess the effect of the altered blood composition in mice with metabolic syndromes on mosquito biology, we allowed mosquitoes to feed directly on 6.66 mg/kg xylazine and 66.6 mg/kg ketamine anesthetized mice [30, 31]. Sugar sources were removed from the mosquito environment the night before offering the mice to ensure a higher propensity for blood feeding. Biological replicates were prepared using independent hatches of mosquitoes and mice from independent dietary protocols.

Groups of mosquitoes, aged between 5 and 6 days, and numbering between 50 and 80 individuals, were allowed to feed for 30 min. After this period, unfed or not fully engorged mosquitoes were identified by visual inspection and were removed from the mosquito cages. Mosquitoes fed on CHOW mice were used as control groups. Mosquito survival was monitored daily for 30 days post blood meal (pbm), and the survival curve was analyzed. Sugar-fed (SF) mosquitoes from the same hatch and age were not allowed to feed on blood and were used as a baseline group. To allow mosquitoes to lay eggs, we placed a moistened paper filter inside the mosquito cages at day 5 pbm. To maintain consistent moisture, the filter paper was positioned on small plastic contained above a column of water to prevent filter drying during the period. The mosquitoes were allowed to lay eggs for 48 h before the filter was removed and dried, and the total mass was weighted [32] and normalized by the total number of mosquitoes present inside the cage at the moment the filter paper was placed.

### Lipid quantification

Following blood feeding on mice, mosquitoes were collected between 1 and 4 days pbm and anesthetized by exposure to cold for 60 s. Mosquitoes were dissected to obtain separated ovaries and abdominal body wall samples, which were further homogenized in PBS using pools of 15 mosquitoes. For the analysis of the triacylglycerol content, the enzyme system Triglycerides 120 (Doles Reagentes^®^, Goiânia, Brazil) was used, according to the manufacturer's protocol; 1 μl homogenized tissues (ovaries and abdominal body wall) were mixed with 200 μl enzyme reagent and incubated at 37 °C for 30 min. Absorbance was measured at 510 nm (Emax^®^Plus-Microplate Reader. Molecular Devices, CA-USA).

### Gene expression

To analyze gene expression, mosquitoes were collected from experimental and control mice at days 1 and 4 pbm, comprising the peak of the blood digestion process and its terminus, respectively [33, 34]. SF mosquitoes from the same hatch were not allowed to feed on blood and were used as a baseline group. SF mosquitoes were dissected and processed on the same day that day 1 pbm mosquitoes were. Briefly, RNA was extracted from the pools of 15–20 whole mosquitoes using the Trizol reagent (Invitrogen) following the manufacturer's protocol. cDNA synthesis was performed using the High-Capacity cDNA Reverse Transcription Kit (Applied Biosystems). Quantitative real-time PCR (qRT-PCR) was performed using SyGreen (Applied Biosystems) and the StepOnePlus Real-Time PCR System (Applied Biosystems). The qRT-PCR cycling conditions were as follows: 95 °C for 10 min, followed by 40 cycles of 95 °C for 15 s, 60 °C for 30 s, and 72 °C for 30 s. Gene expression levels were analyzed using the ∆Ct method, and the data were normalized to the expression level of the housekeeping gene *rp49*. We measured expression of genes of (1) lipid metabolism: fatty acid synthase I (FASI) (AAEL001194), lipid storage droplet surface-binding protein 1 (LSD1) (AAEL005951), lipid storage droplet surface-binding protein 2 (LSD2) (AAEL006820); (2) glucose or energetic metabolism: pyruvate kinase (AAEL014913), glycogen synthase (AAEL004221), PINK1 (AAEL011594); (3) vitellogenesis: apolipophorin (AAEL009955), vitellogenin A1 (AAEL010434); (4) TOR pathway: target of rapamycin (TOR) (AAEL020638); (5) insulin pathway: insulin receptor (AAEL002317), forkhead box O (FoxO) (AAEL019672); (6) epidermal growth factor receptor (EGFR) pathway: Keren (AAEL010067), Vein (AAEL012977); (7) Janus kinase/signal transducers and activators of transcription (JAK/STAT) pathway: signal transducer and activator of transcription (STAT) (AAEL009692), Domeless (AAEL012471), protein inhibitor of activated STAT (PIAS) (AAEL015099); (8) c-Jun N-terminal kinase (JNK) pathway: c-Jun N-terminal kinase (JNK) (AAEL008622 and AAEL008634), Kayak (AAEL008953); (9) cell death/autophagy: autophagy-related protein 8 (ATG8) (AAEL007162); (10) ROS detoxification: catalase (AAEL013407-RB); glutathione peroxidase (AAEL012069); oxidation resistance 1 (AAEL021746); microbiota: Universal 16S. Primer sequences are described in Additional file 1: Table S2.

## Results

### A long-term dietary intervention mimicking Western diets induced insulin resistance and weight gain in mice

**Fig. 1:**
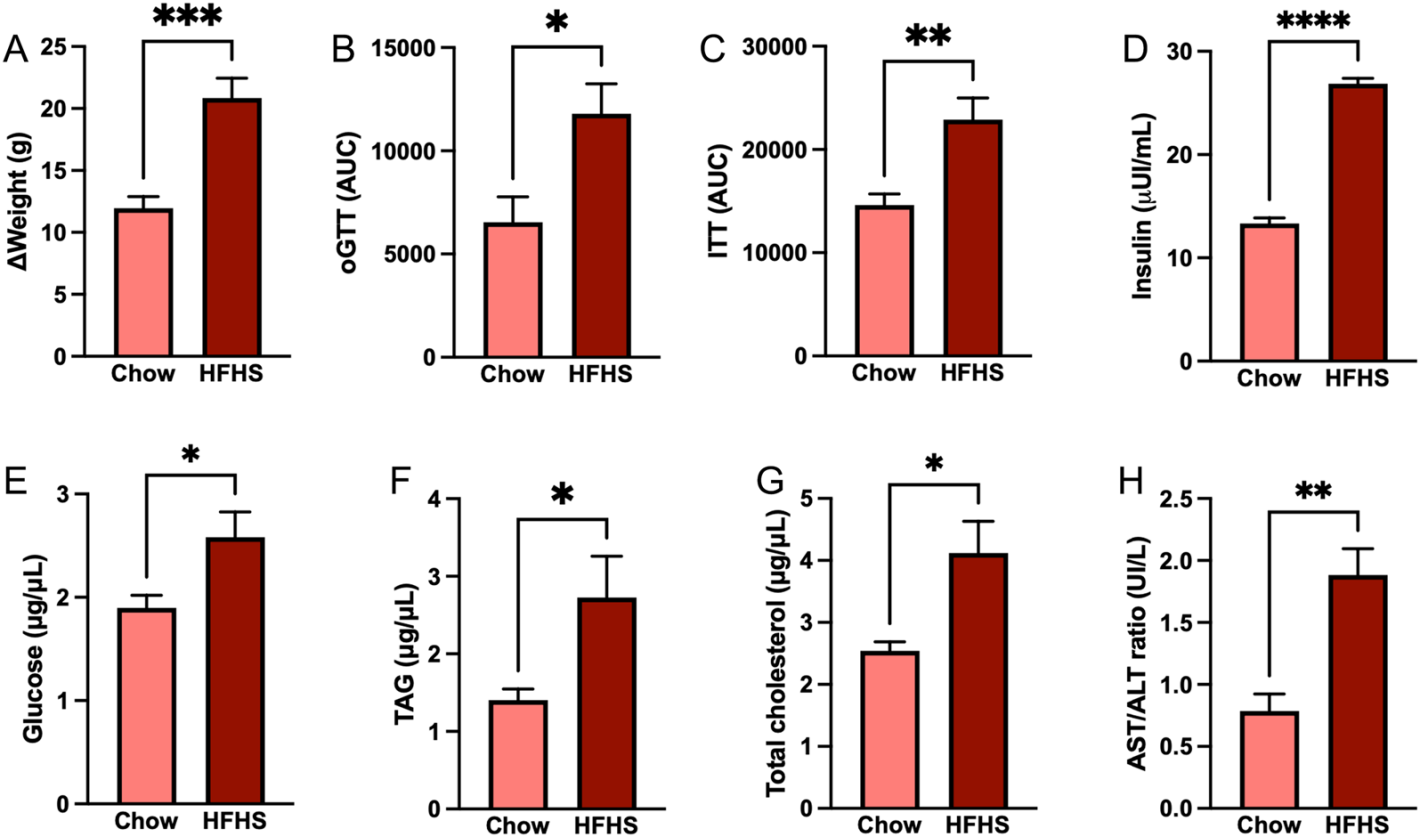
A 20-week HSHF dietary intervention induced metabolic syndrome symptoms in C57 mice. Mice were fed with either a CHOW or an HSHF diet for 20 weeks. Over the course of the experiment, **A** weight gain was monitored weekly, while **B** insulin and **C** glucose tolerance were evaluated in the 19th and 20th weeks, respectively. By the end of the dietary intervention, blood was collected, and **D** insulin, **E** glucose, **F** triglycerides, **G** total cholesterol, and **H** AST/ALT levels were measured. At least six mice were used in each experiment (**A**–**H**). Unpaired t-test was performed. *P < 0.05, **P < 0.01, ***P < 0.001, ****P < 0.0001

To investigate the effect of the Western Diet on *Aedes* mosquito biology, we kept C57Bl6/J mice under an HFHS diet for 20 weeks. We monitored the progression of weight gain and performed glucose tolerance (oGTT) and insulin tolerance (ITT) tests over the course of the dietary intervention. The consumption of the HFHS diet resulted in a significant increase in body weight (11.96 g vs. 20.85 g, *P* < 0.0005) (Fig. 1a). Accordingly, the HFHS diet induced glucose (6542 AU vs. 11,795 AU, *P* < 0.05) (Fig. 1b) and insulin intolerance (14,634 AU vs. 22,901 AU, *P* < 0.01) (Fig. 1c), as detected by glucose clearance following glucose and insulin administration by gavage. To further analyze metabolic alterations produced in mice following an HFHS diet, we quantified metabolites in the blood plasma after the 20-week intervention. Our results show that HFHS mice had increased insulin (13.32 μUI/ml vs. 26.65 μUI/ml, *P* < 0.0001), glucose (1.90 µg/µl vs. 2.58 µg/µl, *P* < 0.05), TAG (1.40 µg/µl vs. 2.72 µg/µl, *P* < 0.05), and cholesterol (2.54 µg/µl vs. 4.12 µg/µl, *P* < 0.05) levels in the blood compared to the CHOW control mice (Fig. 1d–g). Additionally, we found that the aspartate aminotransferase (AST) and alanine aminotransferase (ALT) ratio—a marker for liver damage—was significantly increased in the HFHS group (0.79 UI/l vs. 1.88 UI/l, *P* < 0.01) (Fig. 1h). These findings indicate that an HFHS 20-week dietary intervention successfully induced metabolic changes compatible with those found in metabolic syndrome.

### Blood alterations induced by the HFHS diet result in reduced lifespan and increased egg production and triacylglycerol allocation after mosquito ingestion

**Fig. 2:**
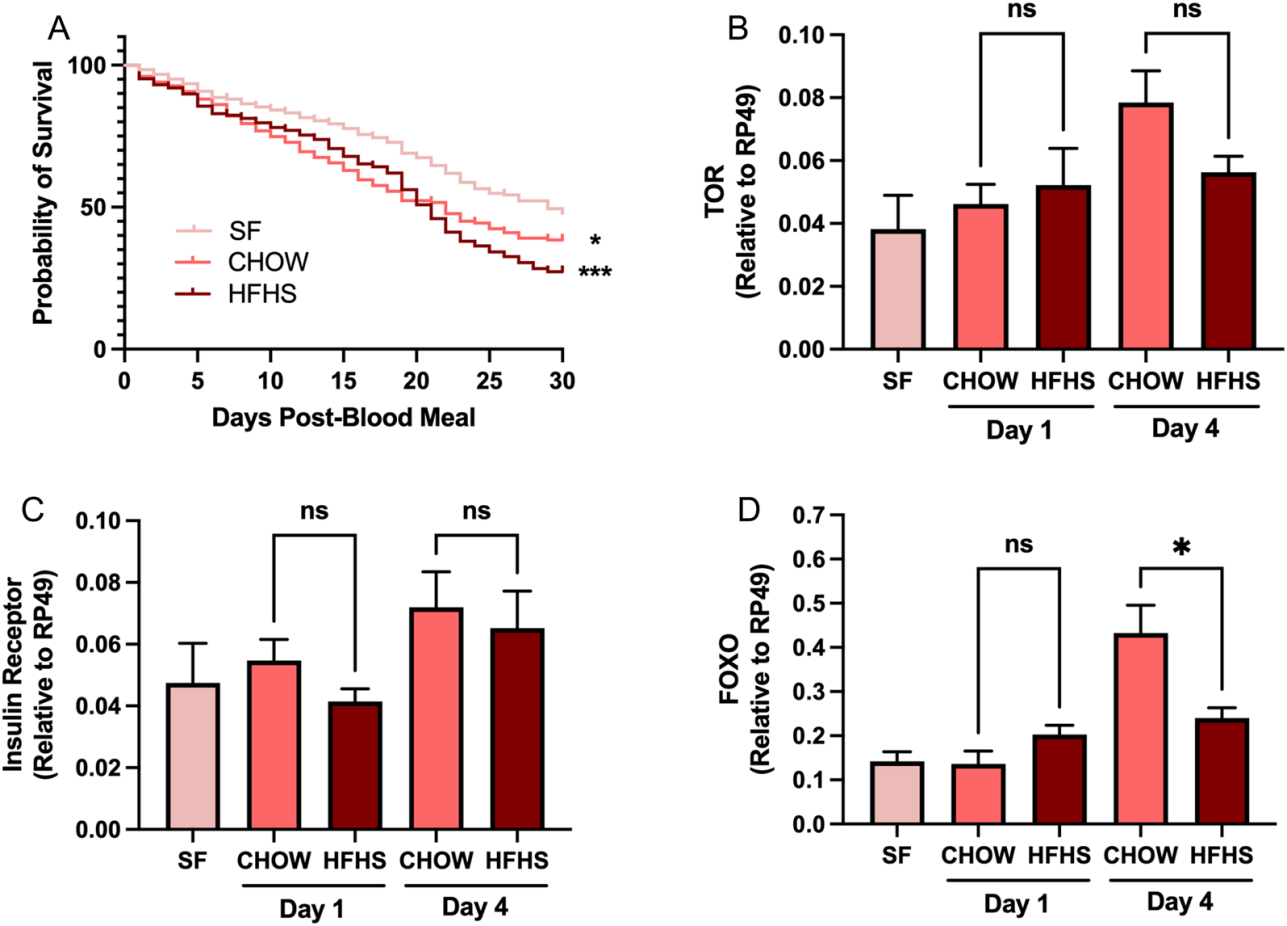
Mosquitoes fed on an HFHS mouse had a reduced survival rate. Mice were fed with either a CHOW or an HSHF diet for 20 weeks. Over the course of the experiment, metabolic syndrome was followed by tracking weight gain, glucose, and insulin sensitivity. Then, *Aedes* mosquitoes were allowed to feed on anesthetized mice, and **A** daily survival was measured over the course of 30 days. Alternatively, whole-body relative expression levels of **B** TOR, **C** insulin receptor, and **D** FoxO was measured by qRT-PCR 1 and 4 days pbm. Four biological replicates were prepared using independent mosquito hatches and dietary protocols. Sugar-fed (SF) mosquitoes were used as a baseline for survival and gene expression. **A** Mantel-Cox test and **B**–**D** one-way ANOVA followed by Tukey’s multiple comparison tests were performed. ns: non-significant, *P < 0.05, ***P < 0.001

We investigated the impact of blood alterations resulting from a Western-type diet on the biology of the yellow fever mosquito *Ae. aegypti* by allowing mosquitoes to feed directly on anesthetized mice. Mosquito survival was daily monitored for 30 days, and the survival curve was analyzed. Blood feeding negatively impacted the 30-day survival rates of mosquitoes compared to sugar feeding (*P* < 0.05). This effect was enhanced when mosquitoes were fed on HFHS-fed mice resulting in an increase in the total number of dead mosquitoes by the end of the period (*P* < 0.001) (Fig. 2a). We looked for differences in the expression of markers of the TOR and insulin pathway, two nutrient-sensing pathways that have a role in the regulation of the aging process [35, 36]. While we could not identify statistical differences in the expression of TOR (Fig. 2b) and insulin receptor (Fig. 2c), we found that forkhead box O (FoxO) (Fig. 2d), a negative regulator of the insulin pathway [37, 38], was downregulated in mosquitoes fed on HFHS blood compared to CHOW-fed mosquitoes 4 days pbm (0.432 vs. 0.240 relative to RP49, *P* < 0.05).

**Fig. 3:**
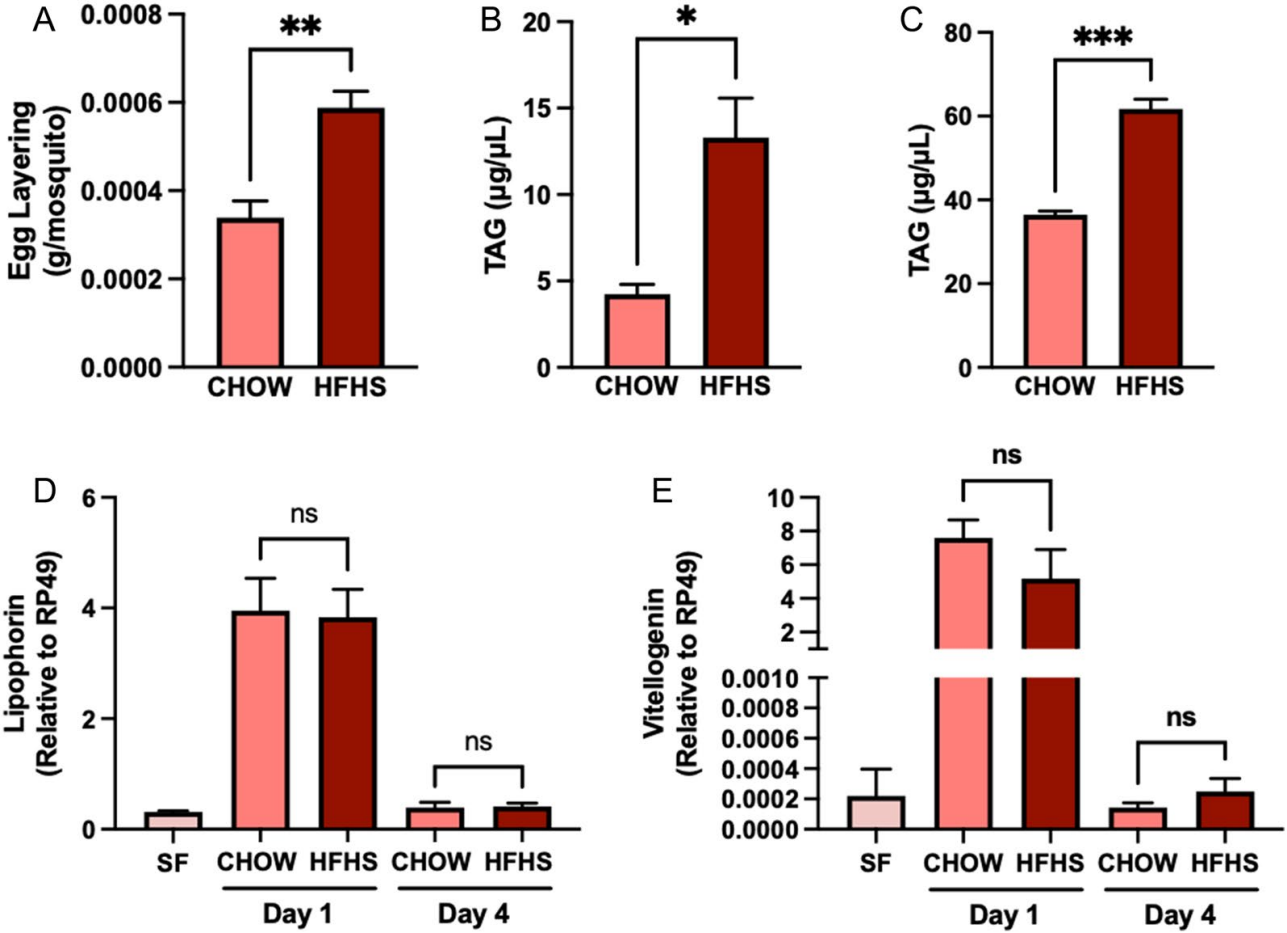
Mosquitoes fed on an HFHS mouse had an increased oviposition and lipid uptake. Mice were fed with either a CHOW or an HSHF diet for 20 weeks. Over the course of the experiment, metabolic syndrome was followed by tracking weight gain, glucose, and insulin sensitivity. *Aedes aegypti* mosquitoes were allowed to feed on anesthetized mice, and **A** egg layering was measured at 5 days pbm. Following a blood meal, mosquitoes were dissected, and TAG content was measured in the **B** fat body and **C** ovaries at 2 and 3 days pbm, respectively. Whole-body relative expression levels of **D** lipophorin and **E** vitellogenin were measured by qRT-PCR 1 and 4 days pbm. Sugar-fed (SF) mosquitoes were used as a baseline for gene expression. Four biological replicates were prepared using independent mosquito hatches and dietary protocols. **A**–**C** Unpaired t-test was performed and **D**–**E** one-way ANOVA followed by Tukey’s multiple comparison tests were performed. ns: non-significant, *P < 0.05, **P < 0.01, ***P < 0.001

We also investigated the impact of mice diet on egg production by *Ae. aegypti* females. For that, we allowed mosquitoes to lay eggs on a filter paper at 5 days pbm. Mosquitoes fed on HFHS mice had an increased reproductive output compared to CHOW-fed mosquitoes (0.00034 g/mosquito vs. 0.00059 g/mosquito, *P* < 0.01) (Fig. 3a). We further dissected mosquitoes fed on either CHOW or HFHS mice to quantify fat body lipid reserves' buildup and mobilization into the ovaries following a blood meal. We found that triacylglycerol (TAG) content in the fat body was increased in mosquitoes fed on HFHS-fed mice compared to CHOW-fed mice 2 days pbm (4.23 µg/µl vs. 13.30 µg/µl, *P* < 0.05) (Fig. 3b). This was followed by an increase in TAG content in the ovaries 3 days pbm (36.50 µg/µl vs. 61.67 µg/µl, *P* < 0.001) (Fig. 3c). Increased egg mass and TAG deposition did not correlate with alterations in the expression of lipophorin (Fig. 3d) and vitellogenin (Fig. 3e)—the major lipid transporters in the hemolymph [17] and the major egg yolk protein [39], respectively. However, feeding on HFHS mice resulted in an apparent early activation of in fatty acid synthase I whole-body expression levels at day 1 pbm, followed by a modest increase in lipid storage protein 2 levels at day 4 pbm (Additional file 1: Fig. S1A–C). We did not detect any major regulation of genes involved in glucose oxidation or metabolism, including pyruvate kinase, glycogen synthase, or PINK1—a marker of mitochondrial turnover (Additional file 1: Fig. S1D–F).

### Blood alterations induced by HFHS-diet on *Ae. aegypti* homeostatic markers

**Fig. 4:**
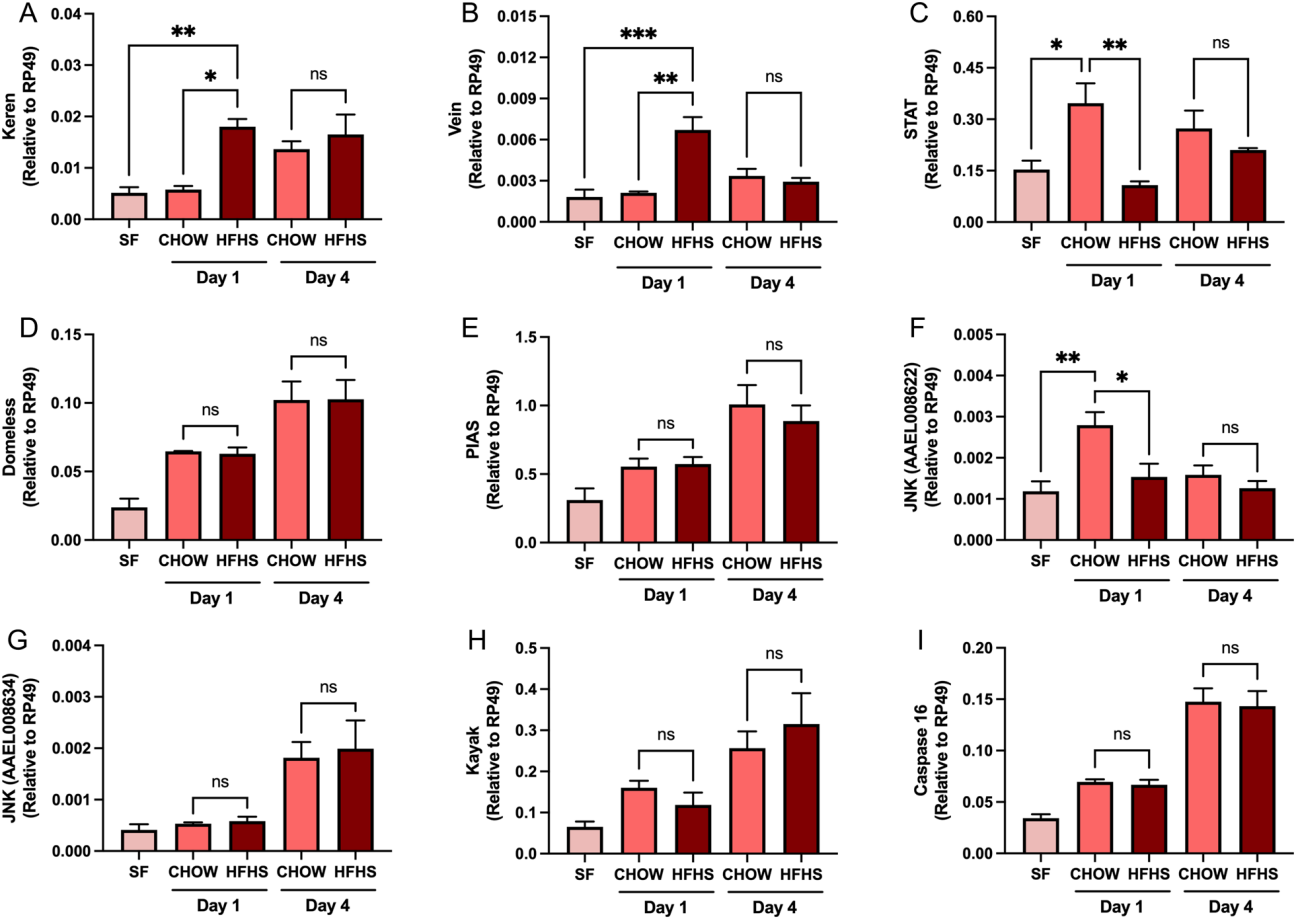
Mosquitoes fed on an HFHS mouse display a discrete regulation of metabolic markers and nutrient-sensing regulators. Mice were fed with either a CHOW or an HFHS diet for 20 weeks. Over the course of the experiment, metabolic syndrome was followed by tracking weight gain, glucose, and insulin sensitivity. Then, mosquitoes were allowed to feed on anesthetized mice, and whole-body relative expression levels of **A** Keren, **B** Vein, **C** STAT, **D** Domeless, **E** PIAS, **F** JNK-AAEL008622, **G** JNK-AAEL008634, **H** Kayak, and **I** caspase 16 were measured by qRT-PCR 1 and 4 days pbm. Sugar-fed (SF) mosquitoes were used as a baseline for gene expression. Four biological replicates were prepared using independent mosquito hatches and dietary protocols. **A**–**I** One-way ANOVA followed by Tukey’s multiple comparison tests was performed. ns: non-significant, *P* < 0.05, **P < 0.01, ***P < 0.001

We aimed to investigate how feeding on HFHS mice impacted mosquito physiology by analyzing different markers of homeostatic regulation at days 1 and 4 pbm—a period comprising the peak of blood digestion and its terminus [33, 34]. We quantified the expression levels of markers of the EGFR pathway—a regulator of cellular proliferation, differentiation, and survival, which has been shown to be activated by insulin following blood feeding in *Aedes* sp. [40] and to play a role in gut regeneration in *Drosophila* [41]*.* We observed an early expression of the *Aedes* homologs of the *Drosophila* EGFR ligand Keren [42] in HFHS mosquitoes at day 1 pbm (0.06 vs. 0.018 relative to RP49, *P* < 0.05) (Fig. 4a). A similar profile was observed with the *Aedes* homologs of the *Drosophila* EGFR ligand Vein [43] (0.002 vs. 0.007 relative to RP49, *P* < 0.01) (Fig. 4b). The EGFR and JAK/STAT pathways have been shown to co-coordinate embryonic development in *Drosophila* [44], and a co-regulation of the JAK/STAT and EGFR pathway has also been implicated in gut regeneration in *Ae. albopictus* [45]. Interestingly, we observed a decrease in STAT—a JAK/STAT pathway transcription factor [46]—in HFHS mosquito day 1 pbm (0.347 vs. 0.108 relative to RP49, *P* < 0.01) (Fig. 4c). However, no difference in expression levels was observed in Domeless and PIAS—the JAK/STAT pathway receptor and its inhibitor, respectively [47] (Fig. 4d, e).

An interplay between EGFR and JNK during *Drosophila* development has recently been shown [48] in keeping with previous descriptions of its coordination in human and other vertebrate models [49, 50]. In *Anopheles*, the malaria vector mosquitoes, the JNK pathway has been shown to participate in the immune response during blood feeding [51] and to be negatively correlated with mosquito longevity [52]. Feeding on HFHS mice resulted in an early suppression of JNK—an activator of the JNK pathway—compared to CHOW-fed mosquitoes (0.003 vs. 0.001 relative to RP49, *P* < 0.05) (Fig. 4f). This early suppression was not observed in other markers of the JNK pathway (Fig. 4g–i), including caspase 16—a marker of cell death in *Aedes* [53] (Fig. 4i). Similarly, we did not detect changes in the total levels of microbiota or reactive oxygen species (ROS) detoxification genes (Additional file 1: Fig. S2), which have been shown to be regulated [54] or regulate the JNK pathway [55] in mosquitoes, respectively.

## Discussion

The worldwide increasing prevalence of metabolic syndrome has been linked to the consumption of Western diets, which are high in saturated fats and simple sugars [2]. Continuous consumption of these diets increases inflammatory cytokines, free fatty acids, glucose, and lipids in blood plasma, which can have serious implications for health, potentially leading to an increased risk of cardiovascular disease and mortality. Mosquitoes, such as the yellow fever mosquito *Ae. aegypti*, rely on blood meals for reproduction and development and are therefore exposed to various components of blood plasma. Previous research has analyzed the effects of individual metabolites, such as insulin and glucose, on mosquito biology and immunity by blood meal supplementation with those components [19, 21, 23]. However, these single-point alterations rarely reflect physiological modulations of blood composition found during metabolic processes.

At present, only a few studies have evaluated the impact of whole-blood composition on mosquito biology. Recently, a correlation between blood from healthy humans and DENV levels in mosquitoes fed from blood from such donors identified free iron levels as a regulator of vector competence [56]. More recently, the impact of an in vivo model of type II diabetes using transgenic mice identified increased susceptibility to Zika virus [57]. These studies did not address the impact on mosquito biology of physiological alterations in total blood composition due to dietary manipulations. To address this gap in knowledge and investigate the impact of Western diets, C57Bl6/J mice were fed an HFHS diet for 20 weeks. By the end of the dietary protocol, mice showed increased weight gain and developed insulin and glucose intolerance, as well as increased levels of circulating insulin, TAGs, and glucose, corroborating that our dietary protocol successfully induced metabolic syndrome symptoms.

Blood digestion is a major challenge for hematophagous insects, which must mitigate free iron and heme toxicity [58–60], build nutritional storage, and coordinate oogenesis and reproduction through nutrient sensing pathways, such as insulin and TOR signaling [61–63]. Previous studies have shown that insulin signaling is a conserved determinant of an organism's life span [64, 65]. Mosquitoes express insulin receptors in the midgut, and artificial insulin supplementation during blood-feeding assays has been shown to negatively impact mosquito longevity [19]. Here, we observed that the HFHS diet resulted in increased plasma insulin levels in mice prior to mosquito feeding. Accordingly, ingestion of HFHS blood by mosquitoes resulted in a stronger reduction of mosquito longevity compared to the ingestion of CHOW blood, in keeping with the hypothesis that increased insulin signaling would reduce mosquito longevity [19]. The observed downregulation of FoxO, a negative regulator of the insulin pathway [38] repressed following insulin receptor/TOR activation [66], might partially explain these findings.

Nutrient sensing nodes play a crucial role in regulating metabolism by detecting changes in the availability of nutrients and signaling appropriate responses to maintain energy balance and mitochondrial function [67]. In that sense, the EGF pathway is an important regulator of PI3K/Akt/mTORC1 [68, 69] in vertebrate models where it plays a critical role in cell proliferation, motility, growth, and differentiation. Insulin and TOR signalings are also key regulators of nutritional allocation and have been shown to regulate egg production following blood feeding [10, 61]. Here, we observed an increase in total egg production following the HFHS diet, in accordance with the early upregulation levels of the EGFR ligands Keren and Vein found in HFHS-fed mosquitoes.

Interestingly, increased egg production did not correlate with a detectable increase in vitellogenin expression. On the other hand, lipid quantification detected that HFHS mice had increased serum TAG and cholesterol levels. Accordingly, mosquitoes fed on HFHS mice had elevated levels of TAG allocation of fat body and ovaries by the time of oogenesis. This pattern correlated with an early increase in the expression of fatty acid synthase and later expression of lipid storage protein 2. Overall, this suggests that the HFHS-induced alterations of blood plasma may impact lipid allocation and metabolism in mosquitoes and compensate for the reduced lifespan by increasing their reproductive output, as they lay significantly more eggs than those fed on control mice. In our experiments, we have used the same cohorts of blood-fed mosquitoes to measure survival and egg-laying, thus minimizing inter-group variability and directly linking survival and fecundity. However, we cannot exclude the possibility that some of the differences observed in total egg mass derive from the alteration of egg size [70] or individual egg mass. Future work should address the impact of different blood-feeding conditions on the correlation between egg number and egg size as well as its impact on egg hatching rates, larval development, and mosquito body size.

The results presented here provide new insights into the impact of blood components on mosquito biology on both biological output and gene expression profiles. Our findings suggest that the Western diet-induced alterations of plasma may have implications for the transmission of infectious diseases by mosquitoes. While we did not detect alteration in the gene expression of stress markers, such as cell death and ROS detoxication, we observed an altered expression of markers of the JNK and JAK/STAT pathways, which have been shown to be important determinators of mosquito immune efficiency and vector competence [40, 51, 71]. The expression of regulators of the EGF pathway, such as Keren and Vein, are likely upstream regulators of such alterations and might have an important role in the modulation of phenotypes observed here.

While further studies will need to address the impact of Western-type diets on *Aedes* immunity and on the extrinsic incubation period of arboviral infections and other pathogens, our present data suggest an impact on vector capacity through signaling pathways that result in altered longevity and fecundity. Reduced lifespan is likely to impact the ability of mosquitoes to transmit diseases during a subsequent blood meal by reducing daily survival rates of mosquitoes fed on such a diet. On the other hand, increased egg production and lipid allocation could result in an increase in mosquito density or larval population resistance. The interplay between metabolic disorders and mosquito-biting behavior also warrants further exploration, particularly as it relates to vector capacity. Mosquitoes are drawn to certain metabolites like CO_2_, lactic acid, and ammonia [72], which are known to be altered in obese or diabetic patients [73–75]. This alteration could feasibly affect the host-seeking behavior of mosquitoes, potentially increasing the propensity of disease vectors to bite affected individuals. The refinement of these variables could allow the refinement of models to correlate the ongoing increase in the prevalence of malnutrition-derived metabolic syndromes with altered vector capacity.

The present study emphasizes the importance of addressing metabolic health to combat the spread of mosquito-borne illnesses and provides a murine model for evaluating the effect of malnutrition on vector biology. Overall, apparent alterations of nutrient-sensing pathways are likely to influence mosquito immunometabolism, an emerging field of study that is only starting to be investigated in insect vectors [76, 77]. Future studies should aim to identify the specific components of blood that are responsible for these effects and to investigate the impact of these alterations on the transmission of mosquito-borne diseases. Overall, this study provides a foundation for further research into the complex interactions between blood components and mosquito biology, which may ultimately lead to the development of new strategies for controlling the spread of mosquito-borne illnesses.

## Conclusions

The rise of metabolic syndromes due to Western diets in various populations has significant ramifications beyond non-communicable diseases, extending to the realm of mosquito-borne disease transmission. Our research highlights that blood composition changes in HFHS diet-fed mice notably impact *Ae. aegypti* biology, with observed trends of decreased survival yet increased egg production. These biological shifts coincide with marked changes in gene expression pertinent to metabolism and nutrient processing. The implications of our study suggest that human dietary patterns in disease-endemic regions may influence vector physiology and disease spread by alterations of vector capacity. Continued research is critical to unravel these complex interactions and to inform disease control measures in the face of changing dietary landscapes.

### Supplementary Information

**Additional file 1: Table S1**. Diet composition. **Table S2**. Primer list. **Figure S1**. Mice were fed with either a CHOW or HSHF diet for 20 weeks. Over the course of the experiment, metabolic syndrome was followed by tracking weight gain, glucose, and insulin sensitivity. Then, *Aedes* mosquitoes were allowed to feed on anesthetized mice, and whole-body relative expression levels of **A** fatty acid synthase I, **B** LSD1, **C** LSD2, **D** pyruvate kinase, **E** glycogen synthase, and **F** PINK1 were measured by qRT-PCR 1 and 4 days pbm. Sugar-fed (SF) mosquitoes were used as a baseline for gene expression. Four biological replicates were prepared using independent mosquito hatches and dietary protocols. **A** Unpaired t-test and **B**–**F** one-way ANOVA followed by Tukey’s multiple comparison tests were performed. *ns*: non-significant, *P < 0.05. **Figure S2**. Mice were fed with either a CHOW or HSHF diet for 20 weeks. Over the course of the experiment, metabolic syndrome was followed by tracking weight gain, glucose, and insulin sensitivity. Then, *Aedes* mosquitoes were allowed to feed on anesthetized mice, and whole-body relative expression levels of **A** 16S, **B** catalase, **C** glutathione peroxidase, and **D** oxidation resistance 1 (**A**–**D**). Sugar-fed (SF) mosquitoes were used as a baseline for gene expression. Four biological replicates were prepared using independent mosquito hatches and dietary protocols. One-way ANOVA followed by Tukey’s multiple comparison tests was performed. *ns*: non-significant.

## Data Availability

All data generated or analyzed during this study are included in this published article.

## Abbreviations

AKT: Protein kinase B
ALT: Alanine aminotransferase
AST: Aspartate aminotransferase
ATG8: Autophagy-related protein 8
EGF: Epidermal growth factor
EGFR: Epidermal growth factor receptor
ERK: Extracellular signal-regulated kinase
FASI: Fatty acid synthase I
FoxO: Forkhead Box O
HFHS: High-fat high-sugar
IGF1: Insulin-like growth factor 1
ITT: Insulin tolerance test
JAK: Janus kinase
JNK: C-Jun N-terminal kinase
LMIC: Low- and middle-income countries
LSD1: Lipid storage droplets surface-binding protein 1
LSD2: Lipid storage droplets surface-binding protein 2
MAPK: Mitogen-activated protein kinase
oGTT: Oral glucose tolerance test
pbm: Post blood meal
PIAS: Protein inhibitor of activated STAT
ROS: Reactive oxygen species
STAT: Signal transducer and activator of transcription
SF: Sugar-fed
TAG: Triacylglycerol
TGFß: Transforming growth factor beta
TOR: Target of rapamycin
