# Supplementary material for: Western diet consumption by host vertebrate promotes altered gene expression on Aedes aegypti reducing its lifespan and increasing fertility following blood feeding
Source: Parasit Vectors. 2024 Jan 6;17:12. doi: 10.1186/s13071-023-06095-3 (PMC10770904; doi:10.1186/s13071-023-06095-3)
Supplement: Supplementary file 1 — Additional file 1: Table S1. Diet composition. Table S2. Primer list. Figure S1. Mice were fed with either a CHOW or HSHF diet for 20 weeks. Over the course of the experiment, metabolic syndrome was followed by tracking weight gain, glucose, and insulin sensitivity. Then, Aedes mosquitoes were allowed to feed on anesthetized mice, and whole-body relative expression levels of A fatty acid synthase I, B LSD1, C LSD2, D pyruvate kinase, E glycogen synthase, and F PINK1 were measured by qRT-PCR 1 and 4 days pbm. Sugar-fed (SF) mosquitoes were used as a baseline for gene expression. Four biological replicates were prepared using independent mosquito hatches and dietary protocols. A Unpaired t-test and B–F one-way ANOVA followed by Tukey’s multiple comparison tests were performed. ns: non-significant, *P < 0.05. Figure S2. Mice were fed with either a CHOW or HSHF diet for 20 weeks. Over the course of the experiment, metabolic syndrome was followed by tracking weight gain, glucose, and insulin sensitivity. Then, Aedes mosquitoes were allowed to feed on anesthetized mice, and whole-body relative expression levels of A 16S, B catalase, C glutathione peroxidase, and D oxidation resistance 1 (A–D). Sugar-fed (SF) mosquitoes were used as a baseline for gene expression. Four biological replicates were prepared using independent mosquito hatches and dietary protocols. One-way ANOVA followed by Tukey’s multiple comparison tests was performed. ns: non-significant. [file 13071_2023_6095_MOESM1_ESM.docx]

Supplemental Information: Western Diet Consumption by Host Vertebrate Promotes Altered Gene Expression on *Aedes aegypti* Reducing its Lifespan and Increasing Fertility Following Blood Feeding

**Supplemental Table 1. Diet Composition.**

**CHOW (AIN93M) Diet**

| Corn Starch | 46.200% |
| --- | --- |
| Casein | 16.500% |
| Dextrinized Starch | 13.000% |
| Sucrose | 10.000% |
| Soybean Oil | 4.000% |
| Microcrystalline Cellulose | 5.000% |
| Mineral Mix specific for AIN 93M diets | 3.500% |
| Vitamin Mix specific for AIN 93M diets | 1.300% |
| L-Cystine | 0.250% |
| Choline Bitartrate | 0.250% |

**High-Fat High-Sucrose Diet**

| Casein | 20.950% |
| --- | --- |
| Sucrose | 27.050% |
| Soybean Oil | 4.000% |
| Microcrystalline Cellulose | 5.000% |
| AIN 93G Mineral Mix | 3.500% |
| AIN 93 Vitamin Mix | 1.300% |
| L-Cystine | 0.300% |
| Choline Bitartrate | 0.250% |
| Lard | 37.650% |

**Supplemental Table 2. Primer List**

| Gene | Description | Forward | Reverse |
| --- | --- | --- | --- |
| - | 16S | TCCTACGGGAGGCAGCAGT | GGACTACCAGGGTATCTAATCCTGTT |
| AAEL009955 | Apolipophorin 2 | AGGCCCGCTTCTTCGATATG | ATGGAAGGCAGCGTTCTTGA |
| AAEL007162 | ATG8/LC3 | TAGTCCCGTCCGACCTAACC | CCGAGTAGAGCGAACCCATC |
| AAEL013407-RB | Catalase | CAATGAACTGCACCGACAAC | AGCCTCATCCAGAACACGAC |
| AAEL012471 | Domeless | CGAAGACTCGCATAGGACCC | CCAGTGCAGAATGACCGAGT |
| AAEL001194 | Fatty acid Synthase I (FASI) | TATGGCCTGATCGGATGTGC | TGGCGATCAACGAACTGGAA |
| AAEL019672 | Forkhead box O  (FoxO) | TCGAAGCGATACGACAGCAG | GTCGGGGGACAGTTGAAAGT |
| AAEL012069 | Glutathione Peroxidase | ACCAGTTCGGGTTCTCAAATG | ACATTGACCCGAGCAAAA AC |
| AAEL004221 | Glycogen Synthase | CACGCCATGGCAAAGGAAAA | GTCAGCACCCTTGTTCGAGA |
| AAEL002317 | Insulin Receptor | GAAGTCATGTGGGGTCGGTT | TTGACGGGCCTCAACACTAC |
| AAEL008622 | JNK | CGTAACGCGAGGGATATGCT | TGACTTCCGCCTCATCCAAC |
| AAEL008634 | JNK | ACCCCGCGGTAGAAATCATC | CCAAACGCTCTTCGCACAAA |
| AAEL008953 | Kayak | TTCCCGTCGTAACATGGGTG | CGAGCTCGTTGGTGTGATCT |
| AAEL010067 | Keren | GTGCCATTGTCGCATTGGTT | CACCGTTCTAAGCACTCCGT |
| AAEL005951 | LSD1 | AACTGATTGCCACCAACCCA | TCGTCAGTAGGACCACCAGT |
| AAEL006820 | LSD2 | GGAGTACGTCGTCACGTTGATTG | GCAGGGGCGGGCTGA |
| AAEL021746 | Oxidation Resistance 1 | ATCCGGGATCCCAATACACT | AGGAGGTTCTCGCTGAACTG |
| AAEL015099 | PIAS | AGCGAACTGATCGAACGTGT | GGAGTCTCTGCTGCTTGCTT |
| AAEL011594 | Pink 1 | GCGCTACCCCCTCGAATAAA | AAACAACAAGATCCGCGTGC |
| AAEL014913 | Pyruvate Kinase | ATACCACGCCAACACGATCA | TCGATCAGCCCAGTACGGAT |
| AAEL009692 | STAT | GGAAAAGAAGGGCACCGAGA | CACCGGAAGTGAGATCGTCC |
| AAEL020638 | TOR | TGGTCAACACGACGACTCAG | GGCTAACTTGACGAGCACCT |
| AAEL012977 | Vein | CCCGATACGTCTACCAACCG | AGCAGTAACGGCGAGTTTGA |
| AAEL010434 | Vitellogenin A1 | GAACTACGTTCGTCCTGGCA | CAGCAGAGGCTTGTACTGCT |
| AAEL003396 | RP49 | GCTATGACAAGCTTGCCCCCA | TCATCAGCACCTCCAGCT |

**Supplemental Figures**

_­_
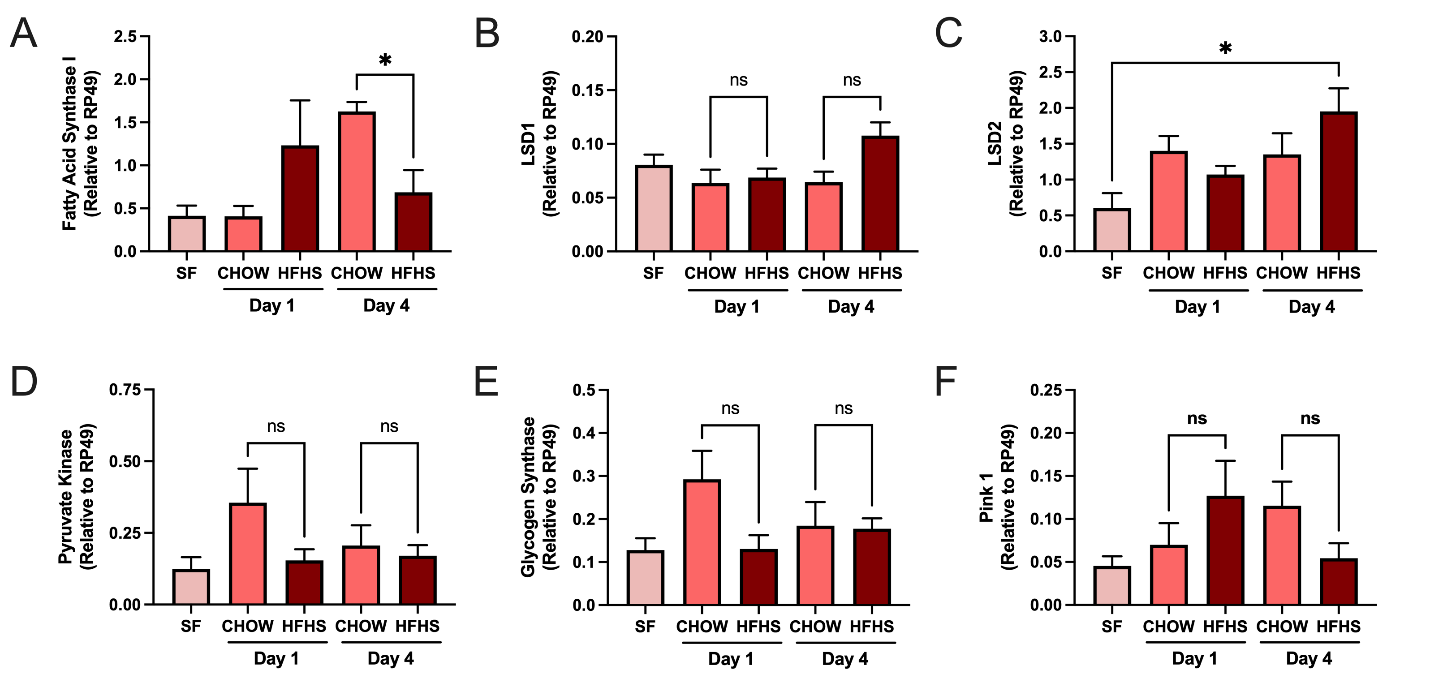


**Additional File 1: Supplemental Figure 1.** Mice were fed with either a CHOW or an HSHF diet for 20 weeks. Over the course of the experiment, metabolic syndrome was followed by tracking weight gain, glucose, and insulin sensitivity. Following, *Aedes* mosquitoes were allowed to feed on anesthetized mice, and whole-body relative expression levels of (A) Fat Acid Synthase I, (B) LSD1, (C) LSD2, (D) Pyruvate Kinase, (E) Glycogen Synthase, (F) Pink1 were measured by qRT-PCR 1 and 4 days pbm. Sugar-fed (SF) mosquitoes were used as a baseline for gene expression. Four biological replicates were prepared using independent mosquito hatches and dietary protocols. (A) Unpaired T-test and (B-F) One-Way ANOVA followed by Tukey’s multiple comparison tests were performed. ns: non-significant, * P <0.05.

**Additional File 2: Supplemental Figure 2.** Mice were fed with either a CHOW or an HSHF diet for 20 weeks. Over the course of the experiment, metabolic syndrome was followed by tracking weight gain, glucose, and insulin sensitivity. Following, *Aedes* mosquitoes were allowed to feed on anesthetized mice, and whole-body relative expression levels of (A) 16S, (B) Catalase, (C) Glutathione Peroxidase, (D) Oxidation Resistance 1 (A-D). Sugar-fed (SF) mosquitoes were used as a baseline for gene expression. Four biological replicates were prepared using independent mosquito hatches and dietary protocols. One-Way ANOVA followed by Tukey’s multiple comparison tests were performed. ns: non-significant.
